# Embelin Suppresses Growth of Human Pancreatic Cancer Xenografts, and Pancreatic Cancer Cells Isolated from Kras^G12D^ Mice by Inhibiting Akt and Sonic Hedgehog Pathways

**DOI:** 10.1371/journal.pone.0092161

**Published:** 2014-04-02

**Authors:** Minzhao Huang, Su-Ni Tang, Ghanshyam Upadhyay, Justin L. Marsh, Christopher P. Jackman, Sharmila Shankar, Rakesh K. Srivastava

**Affiliations:** 1 Department of Pharmacology, Toxicology and Therapeutics, and Medicine, The University of Kansas Cancer Center, The University of Kansas Medical Center, Kansas City, Kansas, United States of America; 2 Department of Biochemistry, University of Texas Health Science Center at Tyler, Tyler, Texas, United States of America; 3 Department of Pathology and Laboratory Medicine, The University of Kansas Cancer Center, The University of Kansas Medical Center, Kansas City, Kansas, United States of America; Florida International University, United States of America

## Abstract

Pancreatic cancer is a deadly disease, and therefore effective treatment and/or prevention strategies are urgently needed. The objectives of this study were to examine the molecular mechanisms by which embelin inhibited human pancreatic cancer cell growth *in vitro*, and xenografts in Balb C nude mice, and pancreatic cancer cell growth isolated from Kras^G12D^ transgenic mice. XTT assays were performed to measure cell viability. AsPC-1 cells were injected subcutaneously into Balb c nude mice and treated with embelin. Cell proliferation and apoptosis were measured by Ki67 and TUNEL staining, respectively. The expression of Akt, and Sonic Hedgehog (Shh) and their target gene products were measured by the immunohistochemistry, and Western blot analysis. The effects of embelin on pancreatic cancer cells isolated from 10-months old Kras^G12D^ mice were also examined. Embelin inhibited cell viability in pancreatic cancer AsPC-1, PANC-1, MIA PaCa-2 and Hs 766T cell lines, and these inhibitory effects were blocked either by constitutively active Akt or Shh protein. Embelin-treated mice showed significant inhibition in tumor growth which was associated with reduced expression of markers of cell proliferation (Ki67, PCNA and Bcl-2) and cell cycle (cyclin D1, CDK2, and CDK6), and induction of apoptosis (activation of caspase-3 and cleavage of PARP, and increased expression of Bax). In addition, embelin inhibited the expression of markers of angiogenesis (COX-2, VEGF, VEGFR, and IL-8), and metastasis (MMP-2 and MMP-9) in tumor tissues. Antitumor activity of embelin was associated with inhibition of Akt and Shh pathways in xenografts, and pancreatic cancer cells isolated from Kras^G12D^ mice. Furthermore, embelin also inhibited epithelial-to-mesenchymal transition (EMT) by up-regulating E-cadherin and inhibiting the expression of Snail, Slug, and ZEB1. These data suggest that embelin can inhibit pancreatic cancer growth, angiogenesis and metastasis by suppressing Akt and Shh pathways, and can be developed for the treatment and/or prevention of pancreatic cancer.

## Introduction

Pancreatic cancer is one of highly aggressive malignant diseases worldwide. The overall 5-year survival rate of this deadly disease is less than 5% [1]. Because of its invasive and metastatic characteristics, <10% of patients are eligible for surgery at the time of diagnosis. The poor prognosis of the disease is related with late presentation, aggressive local invasion, and early metastasis. Conventional chemotherapy and radiotherapy are generally ineffective, and the emergence of drug resistance is common [2], [3]. Gemcitabine is a potent anticancer drug approved for the treatment of pancreatic cancer, but the response rate is very poor. The major deficiencies of current gemcitabine therapy are its rapid metabolic inactivation and narrow therapeutic window. FOLFOX chemotherapy (folinic acid, 5-flurouracil and oxaliplatin) is commonly used for the treatment of pancreatic cancer with limited success. Several factors are associated with increased risk for pancreatic cancer and these include diabetes, chronic pancreatitis, prior gastric surgery, smoking, radiation, and specific gene polymorphisms [4], [5]. Heritable and several acquired gene mutations (e.g. Kras) are common in pancreatic tumors [6]. Mutations in the cyclin-dependent kinase inhibitor p16, the tumor suppressor gene *p53,* and SMAD4 have also been identified [7], [8]. Therefore, understanding the pathogenesis of the preinvasive stage, and developing effective strategies to prevent and/or treat pancreatic cancer are of paramount importance.

Embelin, derived from the fruit of *Embelia ribes* Burm plant (Myrsinaceae), have been shown to possess anticancer activity [9]. Although it was originally discovered as an XIAP inhibitor [10], and it also inhibits cell migration, and invasion and induces apoptosis [11]. It has been shown to induce apoptosis in pancreatic, colon, prostate and lung cancer cells, and chronic leukemia and multiple myeloma cells [12], [13]. It can also modulate tumor-immune microenvironment in Kras^G12D^ mice [14]. Furthermore, STAT3 pathway has been shown to regulate the anti-inflammatory and anti-cancer activities embelin [15]. It enhances the proapoptotic effects of TRAIL [16]. In spite of these findings, the molecular mechanisms by which embelin inhibits tumor growth, angiogenesis and metastasis are not well-understood.

The PI3K/Akt signaling pathway plays significant role in cell proliferation and survival, and it is frequently and aberrantly activated in later stages of pancreatic ductal adenocarcinoma (PDAC) [17]. Pten conditional knockout mice with activated Kras^G12D^ showed significantly accelerated development of acinar-to-ductal metaplasia (ADM), malignant pancreatic intraepithelial neoplasia (mPanIN), and PDAC within 12 months [18]. Most importantly, all mice with Kras^G12D^ activation and Pten homozygous deletion succumbed to cancer by 21 days. This study confirmed the role for PTEN, and the resulting dysregulation of the PI3K/AKT signaling axis in PDAC initiation and progression. Similarly, we have recently demonstrated that resveratrol can inhibit pancreatic carcinogenesis in Kras^G12D^ mice [19]. Here, we sought to examine the anti-proliferative effects of embelin on pancreatic cancer cells isolated from Kras^G12D^ mice.

Sonic hedgehog (Shh) is a member of the Hedgehog (Hh) family of secreted signaling proteins having diverse functions during vertebrate development and in tissue homeostasis [20]. Inappropriate activity of the Hh signaling pathway has been linked to tumor types that arise sporadically or in genetically predisposed individuals [21]. The binding of Shh to Patched (Ptch) receptors causes loss of Ptch activity and consequent phosphorylation and posttranscriptional stabilization of Smoothened (Smo) [22]. The Gli family of transcription factors regulates several genes which paly roles in cell cycle, proliferation, migration and apoptosis [23]. Interestingly, Gli regulates its own expression and other members of Shh pathway such as Patched 1 and Patched 2 and pancreatic cancer cells isolated from Kras^G12D^ mice. The activation of Shh via Smo can occur either by Hh protein stimulation or through loss of Ptch activity [23]. Shh pathway stimulates cell growth in autocrine and paracrine manner [24]. We have recently demonstrated that several chemopreventive agents and anticancer drugs can inhibit pancreatic cancer cell and cancer stem cell growth in vitro and in vivo [19], [25]–[31]. The inhibition of Shh pathway alone or in combination with others can be effective for the treatment and/or prevention of pancreatic cancer.

The purpose of this study was to examine the molecular mechanisms by which embelin inhibits tumor growth, angiogenesis, and metastasis of pancreatic cancer cells xenografted in Balb C nude mice. In addition, the molecular mechanisms by which embelin inhibited growth of pancreatic cancer cells isolated from Kras^G12D^ mice were also examined. Our data showed that embelin inhibited pancreatic cancer cell growth *in vitro,* AsPC-1 xenograft tumor growth *in vivo* and pancreatic cancer cells isolated from Kras^G12D^ mice by suppressing Akt and Shh signaling pathways. In conclusion, it can be developed for the prevention and/or treatment of pancreatic cancer.

## Results

### Embelin Inhibits Cell Viability in Pancreatic Cancer Cell Lines

**Figure 1:**
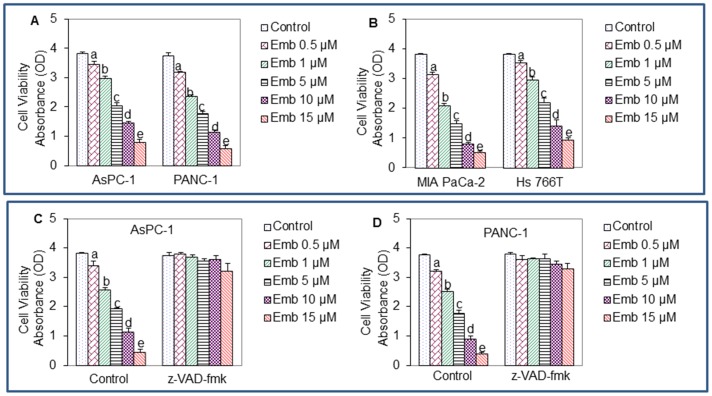
Embelin inhibits cell viability in pancreatic cancer cell lines. (A and B), Pancreatic cancer AsPC-1, PANC-1, MIA PaCa-2 and Hs 766T cell lines were treated with embelin (0–15 μM) for 48 h. At the end of incubation period, XTT assays were performed to measure cell viability. (C and D), Effects of pan caspase inhibitor on anti-proliferative effects of embelin. Pancreatic cancer AsPC-1 and PANC-1 cells were pre-incubated z-VAD-fmk (10 μM) for 2 h and treated with various doses of embelin (0–15 μM) for 48 h. Cell viability was measured by XTT assay. Data represent the mean ± S.D. a, b, c, d, and e = significantly different from respective control, P<0.05.

We first examined the anti-proliferative effects of embelin on four pancreatic cancer cell lines AsPC-1, PANC-1, MIA PaCa-2 and Hs 766T by XTT assay. These cell lines were treated with embelin (0–15 μM) for 48 h, and cell viability was performed by XTT assays. As shown in Fig. 1A and B, embelin inhibited cell viability in all the cell lines. We next examined the involvement of caspase in this process by using a pan caspase inhibitor z-VAD-fmk. Although z-VAD-inhibitor alone was ineffective in inhibiting cell viability, it significantly blocked anti-proliferative effects of embelin on AsPC-1 and PANC-1 cell lines (Fig. 1 C and D). These data suggest that caspase(s) activation may be needed for inhibiting cell growth by embelin.

### Constitutively Active Akt or Shh Protein Inhibits the Anti-proliferative Effects of Embelin

Akt has been shown to regulate Shh pathway by phosphorylating Gli. We next examined whether activation of Akt and Shh pathways blocks anti-proliferative effects of embelin by using constitutively active Akt and Shh protein, respectively. Constitutively active Akt (CA-Akt) has been previously described [28]. Various doses of embelin (0–15 μM) inhibited cell viability of AsPC-1 and PANC-1 cells transfected with empty vector (Fig. 1A and B). Furthermore, constitutively active Akt (CA-Akt) significantly inhibited the anti-proliferative effects of embelin in both the cell lines. These data suggest that embelin can inhibit pancreatic cancer cell proliferation by suppressing Akt pathway.

**Figure 2:**
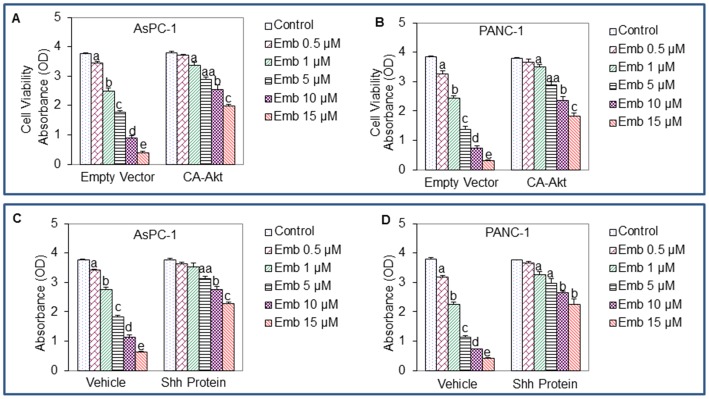
Constitutively active Akt or Shh protein inhibits the anti-proliferative effects of embelin. (A and B), Inhibition of anti-proliferative effects of embelin by constitutively active Akt. Pancreatic cancer AsPC-1 and PANC-1 cells were transiently transfected with either empty vector or constitutively active Akt (CA-Akt), and treated with various doses of embelin (0–15 μM) for 48 h. Cell viability was measured by XTT assay. (C and D), Inhibition of anti-proliferative effects of embelin by Shh protein. Pancreatic cancer AsPC-1 and PANC-1 cells were pretreated with Shh protein (2 μM) for 2 h, and treated with various doses of embelin (0–15 μM) for 48 h. Cell viability was measured by XTT assay. Data represent the mean ± S.D. a, aa, b, c, d, and e = significantly different from each other, P<0.05.

We next examined whether Shh pathway mediates anti-proliferative effects of embelin. Pancreatic cancer cell lines were incubated with Shh protein to activate Gli. As shown in Fig. 2 C and D, various doses of embelin (0–15 μM) inhibited cell viability of AsPC-1 and PANC-1 cell lines. By contrast, Shh protein significantly inhibited the anti-proliferative effects of embelin in these two pancreatic cancer cell lines. These data suggest that embelin can inhibit pancreatic cancer cell proliferation by suppressing Shh pathway.

### Embelin Inhibits the Growth of AsPC-1 Xenografts in Balb C Nude Mice

**Figure 3:**
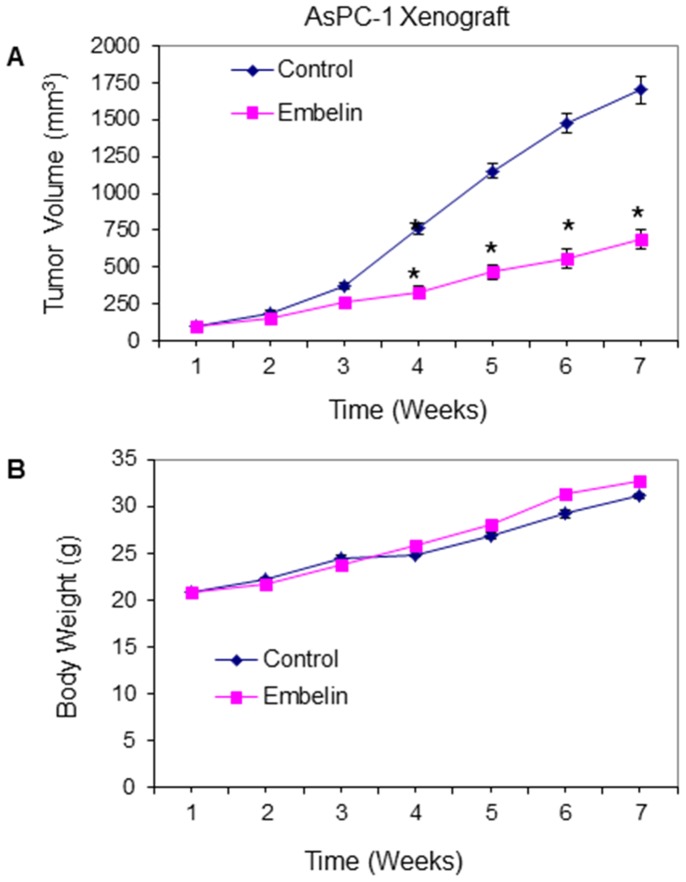
Embelin inhibits the growth of AsPC-1 tumors xenografted in Balb C Nude mice. AsPC-1 cells (2×10^6^ cells mixed with Matrigel, 50∶50 ratio) were subcutaneously implanted into the flanks of Balb C nude mice. Tumor bearing mice were treated with embelin (0 or 40 mg/kg body weight) through gavage (Monday to Friday, once daily) for 6 weeks. Tumor volume (A) and body weight of mice (B) were recorded weekly. Data represent the mean ± S.D. * = significantly different from control, P<0.05.

In order to examine the tumorigenic potential of embelin, we first examined the effects of embelin on growth of AsPC-1 xenografted tumors in Balb C nude mice. AsPC-1 cells were injected subcutaneously into the flanks of Balb C Nude mice. After tumor formation, mice were treated with embelin (0 or 40 mg/kg body weight) through gavage (Monday to Friday, once daily) for 6 weeks. As shown in Fig. 1A, embelin inhibited AsPC-1 pancreatic tumor growth in Balb C nude mice. Furthermore, embelin had no effect on the body weight of AsPC-1 tumor bearing mice, although mice gained weight during the treatment (Fig. 3B). It is important to note that we did not observe any toxicity in the liver, spleen and intestine of mice treated with embelin, suggesting it is a safe natural product.

### Embelin Inhibits Tumor Cell Proliferation, and Induces Apoptosis through Activation of Caspase-3 and Cleavage of Poly (ADP-ribose) Polymerase (PARP)

**Figure 4:**
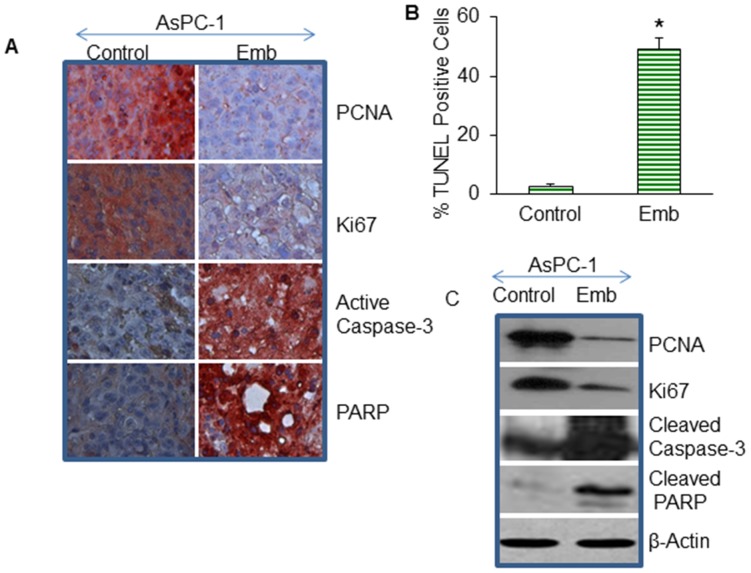
Effects of embelin on cell proliferation and apoptosis. (A), Expression of PCNA, Ki67, caspase-3, and PARP in tumor tissues. Immunohistochemistry was performed to measure the expression of PCNA, Ki67, active caspase-3 and PARP in tumor tissues isolated from control and embelin-treated mice. (B), Quantification of TUNEL positive cells. Apoptosis was measured by TUNEL assay. Data represent the mean ± S.D. * = significantly different from control, P<0.05. (C), Effects of embelin on markers of cell proliferation and apoptosis. Western blot analysis was performed to measure the expression of PCNA, Ki67, caspase-3 and PARP in tumor tissues. The β-actin was used as a loading control.

We next examined the effects of embelin on cell proliferation in tumor tissues derived from control and embelin treated mice using anti-PCNA or anti-Ki67 antibody (Fig. 4). PCNA and Ki67 are the markers of cell proliferation. Embelin inhibited cell proliferation in tumor tissues obtained from AsPC-1 xenografts compared to control mice, as measured by immunohistochemistry (IHC) and the Western blot (WB) analysis.

Caspase activation and cleavage of its substrate PARP are the hall marks of apoptosis [32]. We next examined whether embelin induced tumor cell apoptosis through activation of caspase-3 and cleavage of PARP. Caspase-3 activation was measured by IHC and Western blot analysis using active anti-caspase-3 antibody (Fig. 4A and B). Embelin induced caspase-3 activation. PARP is a substrate of caspase-3 [32]. Embelin treatment resulted in cleavage of PARP. Activation of caspase-3 by embelin correlated with cleavage of PARP in tumor tissues. Overall, these data suggest that embelin inhibited cell proliferation and induced apoptosis in pancreatic tumor tissues through inhibition of PCNA, Ki67, and activation of caspase-3 and cleavage of PARP.

### Embelin Regulates Bcl-2 Family Members and Cell Cycle Proteins, and Inhibits Akt Activation in Tumor Tissues

**Figure 5:**
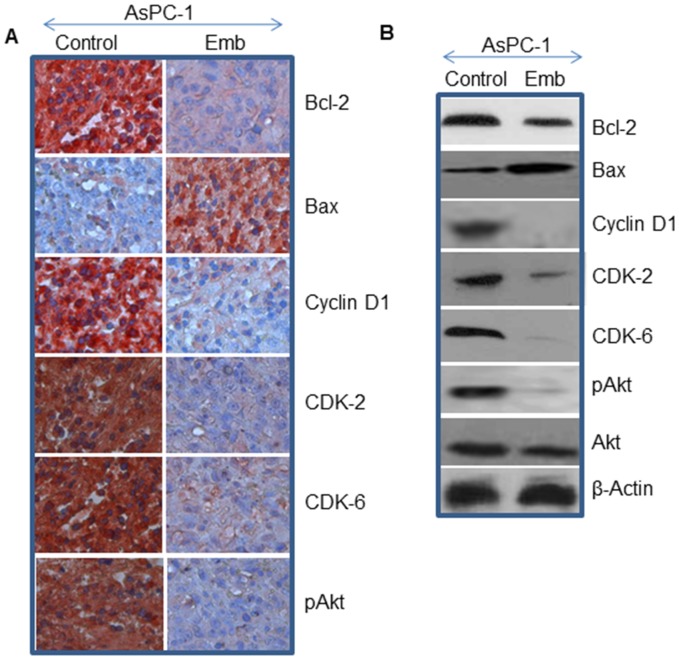
Effects of embelin on the expression of Bcl-2 family members, cell cycle-related proteins and Akt in tumor tissues. (A), Expression of Bcl-2, Bax, Cyclin D1, CDK2, CDK6 and phospho-Akt. Immunohistochemistry was performed as described in material and methods. (B), Western blot analysis was performed to measure the expression of Bcl-2, Bax, Cyclin D1, CDK-2, CDK-6, p-AKT (Ser473), and AKT. The β-actin was used as a loading control.

The members of Bcl-2 family can either promote or inhibit apoptosis [33], [34]. Growth arrest by cell cycle inhibitors can lead to induction of apoptosis [35]. We therefore measured the effects of embelin on the expression of cell cycle proteins (Cyclin D1, CDK-2, and CDK-6), and Bcl-2 family members (Bcl-2, and Bax) in tumor tissues by Western blot analysis and immunohistochemistry. As shown in Fig. 5, embelin inhibited the expression of cell cycle related proteins Cyclin-D1, CDK-2 and CDK-6 in AsPC-1 xenografted tumors compared to untreated control group. Furthermore, embelin treatment of AsPC-1 xenografted mice resulted in inhibition of anti-apoptotic protein Bcl-2 and induction of pro-apoptotic protein Bax in tumors compared to untreated control group. These data suggest that embelin can regulate pancreatic cancer tumor growth by causing cell cycle arrest and inducing apoptosis.

The PI3K/Akt signaling pathway regulates cell cycle progression and tumorigenesis, and is constitutively active in pancreatic cancer [36]. Anticancer agents which inhibit PI3K/Akt pathway can be developed for the management of pancreatic cancer. We therefore measure the expression of phospho-Akt in tumor tissues (Fig. 5). Embelin inhibited the expression of pAkt in tumor tissues isolated from AsPC-1 xenografts compared to untreated control group. Overall, these data suggest that embelin inhibits PI3K/Akt pathway in AsPC-1 xenografted tumors, and inhibition of Akt pathway could induce cell cycle arrest, suppress tumor cell proliferation and pancreatic cancer growth.

### Embelin Inhibits Angiogenesis

**Figure 6:**
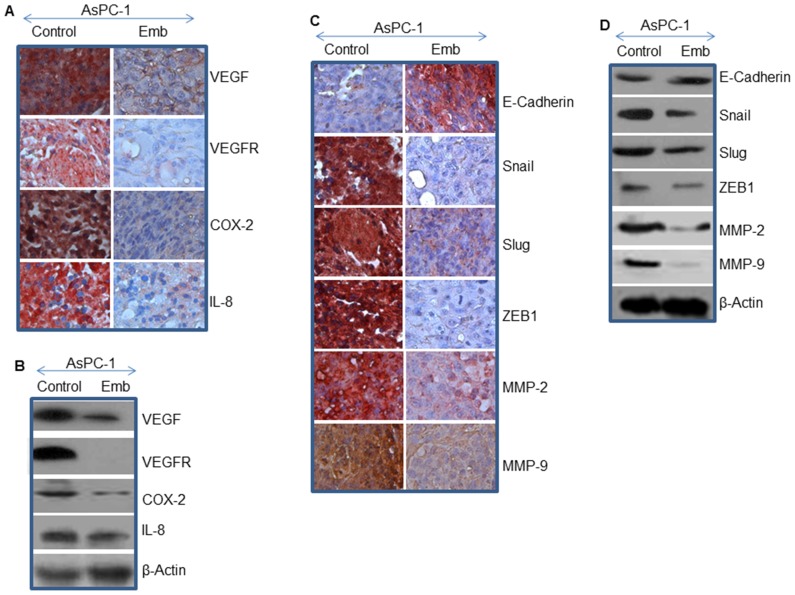
Effects of embelin on markers of angiogenesis, and epithelial-mesenchymal transition. (A), Immunohistochemistry was performed to examine the expression of VEGF, VEGFR, Cox-2, and IL-8 in tumor tissues isolated from control and embelin-treated mice. (B), Western blot analysis was performed to measure the expression of VEGF, VEGFR, Cox-2, and IL-8. The β-actin was used as a loading control. (C), Immunohistochemistry was performed to examine the expression of E-cadherin, Snail, Slug, ZEB1, MMP-2 and MMP-9 in tumor tissues isolated from control and embelin-treated mice. (D), Western blot analysis was performed to measure the expression of E-cadherin, Snail, Slug, ZEB1, MMP-2 and MMP-9. The β-actin was used as a loading control.

The growth of solid tumors depend on angiogenesis for supply of nutrients, growth factors and oxygen [37]. Vascular Endothelial Growth Factor is a secreted growth factor essential for angiogenesis. VEGF functions in both physiological and pathological angiogenesis, particularly in tumor metastasis, making it an attractive therapeutic target. We therefore sought to measure the effects of embelin on angiogenesis by measuring the expression of VEGF and VEGFR in AsPC-1 xenografted tumors. As shown in Fig. 6, treatment of tumor bearing mice with embelin resulted in significant inhibition in VEGF and VEGFR expression in tumor tissues compared to untreated control group.

Cyclooxygenase-2 (COX-2) overexpression promotes inflammation, endothelial cell proliferation, metastasis and tumorigenesis [38]. We therefore examined whether embelin inhibits the expression of COX-2 in AsPC-1 xenografted tumors. As shwon in Fig. 6, embelin inhibited the expression of COX-2 in tumor tissues isolated from AsPC-1 xenografts compared to untreated control group. These data suggest that embelin can suppress inflammation and pancreatic tumor growth by suppressing COX-2.

Cytokines have been implicated in the initiation, progression, and metastasis of solid tumors and angiogenesis [39]. We have recently reported the deregulation of cytokine expression and/or signaling in pancreatic cancer [14], [25], [31]. The IL-8/IL-8 receptor axis plays a crucial role in metastasis and tumor growth, and also modulate tumor microenvironment [39]. We therefore measured the expression of IL-8 in tumor tissues isolated from control and embelin-treated xenografts. Treatment of AsPC-1 xenografted mice with embelin resulted in suppression of IL-8 compared to untreated control group (Fig. 6A and B). These data suggest that inhibition of IL-8/IL-8 receptor axis can be significant in inhibiting pancreatic cancer growth by embelin.

### Embelin Inhibits Markers of Epithelial-to-mesenchymal Transition (EMT) in AsPC-1 Xenografts

Epithelial-to-mesenchymal transition and its reverse process, mesenchymal-to epithelial transition (MET), play important roles in embryogenesis, stemness, cancer progression, metastasis and chemoresistance. Several signaling pathways and regulatory transcriptional networks can regulate EMT [40]. A hallmark of EMT is down-regulation of the cell adhesion molecule E-cadherin, and up-regulation of mesenchymal marker N-cadherin. A variety of transcription factors including the zinc finger Snail homologues (Snail) and basic helix-loop-helix factors such as Twist, ZEB-1, and ZEB2, all interact with the E-box element within the proximal region of the E-cadherin promoter. During EMT, the MMPs digest the extracellular matrix and basement membrane and thus allowing cells to invade and metastasize [41]. We therefore measured the effects of embelin on the expression of E-cadherin, Snail, Slug, ZEB1, MMP-2 and MMP-9 in tumor tissues. Treatment of AsPC-1 xenografted mice with embelin induced the expression of E-cadherin and inhibited the expression of MMP-2, MMP-9, Snail, Slug, and Zeb-1 in tumor tissues compared to untreated control group (Fig. 6 C and D). Our data demonstrate that embelin can inhibit/reverse pancreatic tumor metastasis by inducing the expression of E-cadherin and inhibiting its associated transcription factors (Snail, Slug, and ZEB1) and MMPs (MMP-2 and MMP-9). Overall, our data demonstrate that embelin can a potential inhibitor of early metastasis.

### Embelin Inhibits Sonic Hedgehog Pathways, and Up-regulates TRAIL-R1/DR4 and TRAIL-R2/DR5

**Figure 7:**
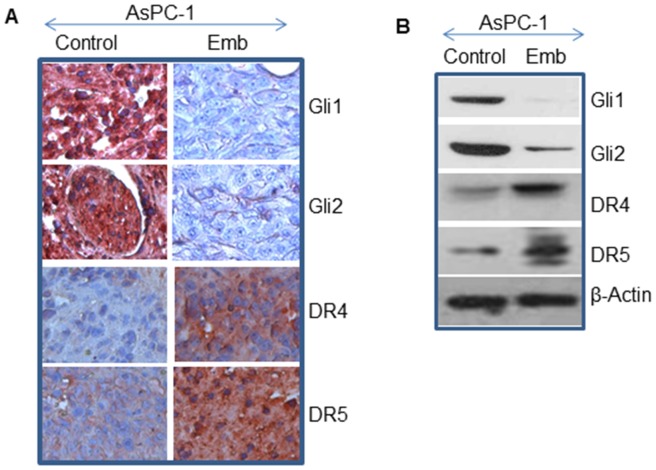
Effects of embelin on the expression of Sonic hedgehog pathways, and TRAIL-R1/DR4 and TRAIL-R2/DR5. (A), Immunohistochemistry was performed to measure the expression of Gli1, Gli2, DR4 and DR5 in tumor tissues isolated from control and embelin-treated mice. (B), Western blot analysis was performed to measure the expression of Gli1, Gli2, DR4 and DR5. The β-actin was used as a loading control.

Shh pathway promotes cell invasion, migration, metastasis, and tumor growth by mediating a complex signaling network in pancreatic cancer [20], [42]. Inhibition of Shh pathway has been shown to suppress tumor growth and metastasis. We therefore sought to examine the effects of embelin on Shh pathway by measuring the expression of transcription factors Gli1 and Gli2. Gli1 regulates its own expression. Treatment of AsPC-1 xenografted mice with embelin inhibited the expression of Gli1 and Gli2 compared to untreated control (Fig. 7 A and B). These data suggest that embelin can inhibit AsPC-1 tumor growth by suppressing Shh pathway.

We have demonstrated that the activation of TRAIL-death receptor pathway induces apoptosis in cancer cells [43]–[45]. Since TRAIL-R1/DR4 and TRAIL-R2/DR5 are induced by the inhibition of Gli activity [29], Shh inhibitors can be combine with the ligand TRAIL to induce apoptosis. We therefore examined the effects of embelin on the expression of TRAIL-R1/DR4 and TRAIL-R2/DR5 in tumor tissues isolated from AsPC-1 xenografts (Fig. 7 A and B). Treatment of AsPC-1 tumor bearing mice with embelin up-regulated the expression of TRAIL-R1/DR4 and TRAIL-R2/DR5 in tumor tissues compared to untreated control group. These data suggest that embelin can be combined with death receptor ligands (TRAIL or agonistic antibodies) for the treatment of pancreatic cancer.

### Embelin Inhibits Growth of Pancreatic Cancer Cells Isolated from Kras^G12D^ Mice

**Figure 8:**
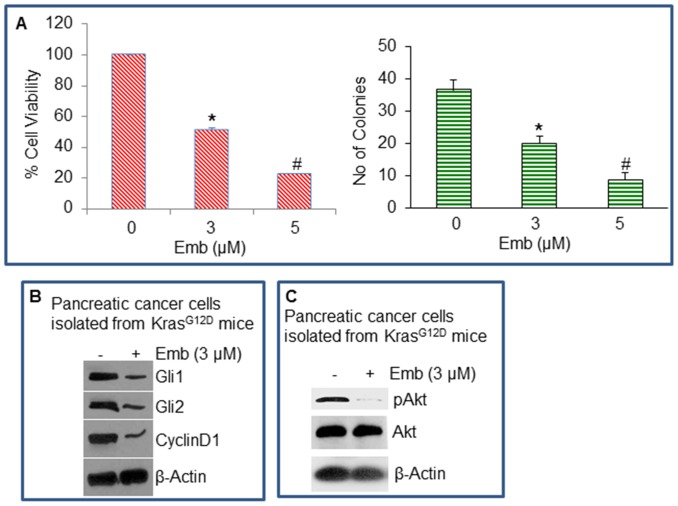
Effects of embelin on cell viability and colony formation, and Shh and Akt pathways in pancreatic cancer cells isolated from Kras^G12D^ mice. (A), Pancreatic cancer cell were isolated from 10-months old Kras^G12D^ mice. Cells were treated with embelin (0–5 μM) to measure cell viability and colony formation at 2 and 21 days, respectively. * = significantly different from control, P<0.05. (B), Mouse pancreatic cancer cells were isolated from 10 months old Kras^G12D^ mice, and treated with or without embelin (3 μM) for 36 h. At the end of incubation period, crud protein was extracted. Western blot analysis was performed to measure the expression of Gli1 and Gli2. The β-actin was used as a loading control. (C), Mouse pancreatic cancer cells were isolated from 10 months old Kras^G12D^ mice, and treated with or without embelin (3 μM) for 36 h. Western blot analysis was performed to measure the expression of phopho-Akt (pAkt) and Akt. The β-actin was used as a loading control.

Kras mutations are found in approximately 95% of human pancreatic ductal adenocarcinomas [6]. We therefore examined the effects of embelin on growth characteristics and signaling pathways in mouse pancreatic cancer cells isolated from 10-months old Kras^G12D^ mice. Pancreatic cancer cells were isolated from mice and *in vitro* studies were performed to examine the biological effects of embelin. Embelin inhibited cell viability and colony formation in mouse pancreatic cancer cells (Fig. 8A). In order to confirm the role of Shh, and Akt pathways on anti-proliferative effects of embelin, we measured the expression of components of these pathways. Embelin inhibited the expression of Gli1 and Gli2 and their down-stream target Cyclin D1 in mouse pancreatic cancer cells (Fig. 8B). Furthermore, embelin inhibited the expression of phospho-Akt, a kinase highly active in pancreatic cancer (Fig. 8C). These data suggest that embelin can inhibit mouse pancreatic cancer growth by suppressing Shh and Akt signaling pathways.

## Discussion

Pancreatic cancer is one of the most aggressive and devastating malignancies. We have demonstrated, for the first time, that embelin inhibited viability of pancreatic cancer cell lines *in vitro* and AsPC-1 xenografted tumor growth which was associated with suppression of Akt and Shh pathways. Furthermore, embelin inhibited the growth of pancreatic cancer cells isolated from Kras^G12D^ mice through suppression of Akt and Shh pathways. These pathways have been shown to play major roles in pancreatic carcinogenesis. Embelin inhibited tumor cell proliferation, and cell cycle, and induced apoptosis. Embelin also inhibited markers of angiogenesis and metastasis. Interestingly, treatment of AsPC-1 xenografted mice with embelin resulted in up-regulation of death receptor DR4 and DR5, suggesting the combination of embelin with TRAIL agonists could be a viable strategy to treat human pancreatic cancer.

The PI3K/Akt signaling pathway regulates cell proliferation and survival, and is frequently and aberrantly activated in PDAC. In our study, embelin inhibited the phosphorylation/activation of Akt in human and mouse pancreatic cancer cells and tissues. Activation of Kras results in phosphorylation and activation of Akt kinase. Since embelin induced apoptosis in pancreatic cancer cells harboring Kras mutation by suppressing Akt pathway, suggesting its clinical benefits against human pancreatic cancer where Kras is mostly mutated. In a recent study, the heterozygous loss of Pten in Kras^G12D^ mutant mice accelerated the development of acinar-to-ductal metaplasia (ADM), mPanIN, and PDAC within one year [18]. This study strongly suggests the role of PTEN/PI3K/Akt and Kras signaling pathways in both pancreatic cancer initiation and progression.

Shh is abnormally expressed in pancreatic adenocarcinoma and its precursor lesions (PanIN). Pancreata of Pdx-Shh mice (in which Shh is misexpressed in the pancreatic endoderm) develop abnormal tubular structures, PanIN-1 and -2 [46]. Moreover, these PanIN lesions also contain mutations in K-ras and overexpress HER-2/neu, which are genetic mutations found early in the progression of human pancreatic cancer. We have recently demonstrated that the components of Shh pathway are highly expressed in human pancreatic cancer stem cells and pancreatic cancer cell lines, and several chemopreventive agents inhibited pancreatic cancer growth [19], [26], [27], [47]. Similarly in the present study, embelin inhibited AsPC-1 tumor growth and mouse PDAC cell growth by suppressing Shh pathway. In another study, it was demonstrated that inhibition of the Hh pathway decreased cell proliferation and induced apoptosis through inhibition of the PI3K/Akt pathway and cancer stem cells [48]. Furthermore, we have demonstrated that inhibition of the Shh signaling pathway significantly inhibited EMT by suppressing the activation of transcription factors Snail and Slug, which were correlated with significantly reduced pancreatic cancer stem cell invasion [26], [27], [47], [49], [50], suggesting that the Shh signaling pathway is involved in early metastasis. Overall, these data suggest that inhibition of the Shh pathway may be a potential molecular target of new therapeutic strategies for human pancreatic cancer.

Accumulating evidence suggests an important role for COX-2 in the pathogenesis of a wide range of malignancies. COX-2 is upregulated in pancreatic PDAC [38]. COX-2 deletion in Pdx1+ pancreatic progenitor cells significantly delays the development of PDAC in mice with K-ras activation and Pten haploinsufficiency. Conversely, COX-2 overexpression promotes early onset and progression of PDAC in the K-ras mouse model. Loss of PTEN function is a critical factor in determining lethal PDAC onset and overall survival. Mechanistically, COX-2 overexpression increases p-Akt levels in the precursor lesions of Pdx1(+); K-ras(G12D)(/+); Pten(lox)(/+) mice in the absence of Pten LOH. In contrast, COX-2 deletion in the same setting diminishes p-Akt levels and delays cancer progression. This study suggests an important cell intrinsic role for COX-2 in tumor initiation and progression through activation of the PI3K/Akt pathway. In the absence of intrinsic COX-2, PDAC eventually develops with decreased FKBP5 and increased GRP78 expression, two alternate pathways leading to Akt activation [38]. Therefore, simultaneous inhibition of both COX-2 and Akt may represent a novel strategy for the management of pancreatic cancer.

Tumor cells undergoing EMT are also known to increase the secretion of specific factors, including cytokines, chemokines and growth factors, which could play an important role in tumor progression [39], [51]. In the present study, embelin inhibited the expression of IL-8 in AsPC-1 tumor tissues. Thus, IL-8 signaling blockade by embelin may provide a means of inhibiting or reversing EMT. Furthermore, inhibition of Snail, Slug and ZEB-1 expression and upregulation of E-cadherin by embelin could regulate pancreatic cancer progression through its influence on reversal of EMT. Accordingly, inhibition of the expression or function of EMT-inducing transcription factors in pancreatic cancer is anticipated to lead to new therapeutic strategies.

Cancer cell metastasis is a step-wise process that includes detachment of cells from the primary tumor, local proteolysis of the basement membrane, intravasation, survival of the circulation, arrest in distant organ, extravasation and invasion into the surrounding tissue and growth. Metastasis involves penetration of the ECM and basement membrane, and requires the action of proteases (MMPs) [40]. We have recently demonstrated that embelin can inhibit pancreatic cancer growth in Kras^G12D^ mice by modulating tumor-immune microenvironment [14]. Specifically CTL, NKT, γδT, NK, and IFNγ (Th1 type) cells were up-regulated, and Th17, PMN-MDSC, IL-6 and IL-8 (Th2 type) immune cells were inhibited [14], suggesting embelin can inhibit pancreatic cancer growth and inflammation by modulating tumor immune microenvironment. Our studies suggest that embelin can inhibit pancreatic tumor growth by regulating angiogenesis and metastasis.

## Conclusions

Our study provides important information regarding the antitumor activities of embelin in human and mouse pancreatic cancer. Specifically, we have demonstrated that embelin inhibited human pancreatic cancer cell viability *in vitro* and AsPC-1 xenografted tumor growth by suppressing Akt and Shh pathways. Embelin inhibited the production of pro-angiogenic IL-8 and VEGF/VEGFR as well as invasiveness-promoting MMP-2 and MMP-9 thus blocking production of tumorigenic mediators in the microenvironment of the tumor. Furthermore, embelin inhibited mouse pancreatic cancer growth in Kras^G12D^ mice by suppressing Akt and Shh pathway. The up-regulation of TRAIL-R1/DR4 and TRAIL-R2/DR5 by embelin suggests a potential therapeutic benefit of combining it with the death receptor agonists. Our studies suggest that inhibition of Akt and Shh pathways by embelin act together to suppress pancreatic cancer growth. Thus, embelin can be used for the treatment and/or prevention of pancreatic cancer.

## Materials and Methods

### Reagents

Antibodies against phospho-Akt, Akt, Gli1, Gli2, cyclin D1, CDK-2, CDK-6, PCNA, Ki67, caspase-3, PARP, Bcl-2, Bax, Cox-2, VEGF, VEGFR, MMP-2, MMP-9, TRAIL-R1/DR4, TRAIL-R2/DR5, E-Cadherin, Snail, Slug, ZEB1 and β-actin were purchased from Cell Signaling Technology, Inc. (Danvers, MA). Shh protein was purchased from Abcam (Cambridge, MA). Pan caspase inhibitor z-VAD-fmk was purchased from Calbiochem/Millipore. Embelin was purchased from LKT Laboratories, Inc. (St. Paul, MN).

### Trypan Blue Assay

Mouse pancreatic cancer cells (1×10^4^) were incubated with 0, 3, and 5 μM of embelin in 1 ml of RPMI 1640 medium in 6-well plate for 48 h. At the end of incubation period, cell viability was determined by the trypan blue assay.

### XTT Assays

Cells (1.5×10^4^) were incubated with embelin in 250 μl of RPMI 1640 medium in 96-well plate for 48. Cell viability was determined by the XTT assay. During the assay, the yellow tetrazolium salt XTT is reduced to a highly colored formazan dye by dehydrogenase enzymes in metabolically active cells. This conversion only occurs in viable cells and thus, the amount of the formazan produced is proportional to viable cells in the sample. In brief, a freshly prepared XTT-PMS labeling mixture (50 μl) was added to the cell culture. The absorbance was measured at 450 nm with.

### Antitumor Activity of Embelin

Animal protocol (number 372) was approved by the Institutional Animal Care and Use Committee (IACUC) of the University of Texas Health Science Center at Tyler, Tyler, Texas. The institutional and national guidelines for the care and use of animals were followed.

AsPC-1 cells (1×10^6^ cells mixed with Matrigel, Becton Dickinson, Bedford, MA, 50∶50 ratio, in a final volume of 75 μl) were injected subcutaneously into the flanks of Balb/c *nu/nu* mice (4–6 weeks old). Balb C Nude mice were purchased from the National Cancer Institute, Frederick, MD. After tumor formation, mice (7 mice per group) were treated with embelin (0 or 40 mg/kg body weight) through gavage (Monday to Friday, 5 days a week for 6 weeks, once daily). At the end of the experiment, mice were euthanized and tumors were isolated, weighed and biochemically analyzed.

We have described the generation of Kras^G12D^ mice elsewhere [19]. LSL K-ras^G12D^ and Pdx-1-Cre mice were obtained from the National Cancer Institute (Frederick, MD). LSL K-ras^G12D^ mice were crossed with the Pdx-1-Cre mice to obtain *Kras^G12D^ (Pdx1-Cre;LSL-Kras^G12D^)* mice as described [19]. The recombined Kras*^G12D^* allele was identified by PCR. *Pdx1-Cre;LSL-Kras^G12D^* mice developed early stage mPanIN lesions at 2 months of age, and at this age the vast majority of ducts were normal [52]. Mice developed significant numbers of advanced mPanIN lesions (stages 2 and 3) at about 6 months, and the vast majority of ducts were abnormal [52]. Kras*^G12D^* mice began to develop invasive and metastatic pancreatic ductal adenocarcinoma after 6 months of age. We have isolated pancreatic cancer cells from 10-months old Kras^G12D^ mice. Mouse pancreatic cancer cells were treated *in vitro* with embelin to examine its effects on cell growth, colony formation and Akt and Shh pathways.

### Western Blot Analysis

Western blots were performed as we described earlier [27].

### Immunohistochemistry and TUNEL Assay

Imunohistochemistry of tumor tissues collected was performed as we described elsewhere [27]. TUNEL assays were performed as per manufacturer’s instructions (Roche Applied Sciences).

### Statistical Analysis

The mean and SD were calculated for each experimental group. Differences between groups were analyzed by one or two way ANOVA, followed by Bonferoni’s multiple comparison tests using PRISM statistical analysis software (GrafPad Software, Inc., San Diego, CA). Significant differences among groups were calculated at P<0.05.
